# Japanese Clinical Physical Therapists With the Mechanical Diagnosis and Therapy License Are More Competent and Confident in Pain Management Than Those Without It: A Cross-Sectional Study

**DOI:** 10.7759/cureus.70652

**Published:** 2024-10-01

**Authors:** Hiroshi Takasaki, Takahiro Ueno

**Affiliations:** 1 Department of Physical Therapy, Saitama Prefectural University, Koshigaya, JPN; 2 Department of Rehabilitation, Koshigaya Rehabilitation Care Center, Koshigaya, JPN

**Keywords:** biopsychosocial model, clinical practice guideline, evidence-based practices, mckenzie method, musculoskeletal physiotherapeutic management, pain knowledge, pain management, post-graduate medical education\, self-confidence, treatment decision-making

## Abstract

Background

The McKenzie Method of Mechanical Diagnosis and Therapy (MDT) has long been misunderstood as a biomedical approach. In fact, it is a biopsychosocial approach with an up-to-date evidence-based educational curriculum. Recently, it has become possible to partially measure competence in clinical decision-making based on contemporary pain understanding and confidence in pain management using the Pain Understanding and Confidence Questionnaire (PUnCQ). The primary aim of this study was to compare the following outcomes between physical therapists with and without credential license in MDT (Cred.MDT) and the PUnCQ as well as attitudes toward the biopsychosocial perspective, adherence to evidence-based management for low back pain (LBP), and knowledge of modern pain science. The secondary aim was to explore relevant factors in the PUnCQ.

Methodology

Clinical physical therapists who were managing patients with pain were recruited from two associations (the Japanese Society of Allied Health and Rehabilitation and the Japan Branch of the International McKenzie Institute, who had all acquired at least the Cred.MDT). The following outcomes were measured: (1) the PUnCQ-1 for partial competence in evidence-based clinical decision-making for pain management; (2) part 2 scores of the PUnCQ for confidence in pain management; (3) the Pain Attitudes and Beliefs Scale for Physical Therapists (PABS-PT) biopsychosocial/biomedical ratio for treatment perspectives; (4) the Knowledge and Attitudes of Pain (KNAP) for knowledge of modern pain science; and (5) a questionnaire for adherence to LBP practice guidelines. Two group comparisons were conducted for the primary aim and a multiple regression analysis for the independent variable of the PUnCQ-1 was conducted for the secondary aim.

Results

Data from 122 physical therapists (63 and 59 participants with and without the Cred.MDT, respectively) were analyzed. Statistically significantly higher scores were detected for physical therapists with Cred.MDT compared to those without (all p<0.05) for all of the above outcomes. The multiple regression analysis demonstrated that statistically significant contributors to the PUnCQ-1 were part 2 scores of the PUnCQ for the pain management factor (p = 0.016) and acquisition of the Cred.MDT (p = 0.038) (R^2^ = 0.12).

Conclusion

Competence and confidence in pain management, attitudes toward biopsychosocial approaches, knowledge of modern pain science and guideline adherence are higher in physical therapists with the Cred.MDT than those without it. Confidence in pain management and acquisition of the Cred.MDT contributed to competence in evidence-based clinical decision-making for pain management.

## Introduction

Post-graduate education courses for musculoskeletal physical therapy often lack supporting evidence [1], which may be one reason why evidence-based practice (EBP) has not been widespread in physical therapy [2]. Therefore, post-graduate clinical training programs that are constantly updated are recommended [1], and it is important to identify which post-graduate clinical training programs would contribute to the improvements in EBP competence in clinical physical therapists and achieve best clinical outcomes efficiently, particularly for countries with low EBP competence and attitude, such as Japan [3,4].

The McKenzie method of Mechanical Diagnosis and Therapy (MDT) is one of the established post-graduate clinical training programs of musculoskeletal physical therapy with an up-to-date evidence-based educational curriculum and standardized written and practical examinations all over the world. The level of MDT competence has been proven to change the reliability [5] of identifying treatment effect modifiers that lead to clinical decision-making such as centralization and directional preference [6] and may make a difference in the effectiveness of treatment. MDT has continued to be updated since its development. When the educational manuals are updated, the contents are peer-reviewed by researchers in the relevant fields. MDT has long been misunderstood as a biomedical approach. In fact, it is a biopsychosocial approach that is based on various research findings including biopsychosocial aspects. In MDT, the treatment strategy is guided by identifying the MDT classification, but even within that classification, specific intervention strategies vary in consideration of the biopsychosocial pain drivers [7]. It has been reported that psychological status actually improves after an MDT intervention [8,9].

A previous study showed that practitioners with the MDT credential license (Cred.MDT) adhere more to EBP guidelines of low back pain (LBP) and have a more biopsychosocial perspective on the management of patients with LBP than general physical therapists in Japan [10]. Attitudes toward the biopsychosocial perspective are known to correlate with knowledge of pain physiology [11]. In fact, pain neuroscience education is used in contemporary MDT intervention [12]. Therefore, it is hypothesized that clinical physical therapists with the Cred.MDT have greater knowledge of modern pain science than clinical physical therapists without the Cred.MDT, but no study has proven this hypothesis.

Furthermore, recently it has become possible to measure partial clinical competence in decision-making based on contemporary pain understanding using the Pain Understanding and Confidence Questionnaire (PUnCQ) [13]. The PUnCQ has been developed [14,15] based on the core curriculum of the International Association for the Study of Pain [16] and the British Pain Society [17]. The first part of the PUnCQ (PUnCQ-1) probes 12 aspects of pain management for a clinical vignette featuring whiplash-associated disorders and indicates partial clinical competence in evidence-based decision-making for pain management; the second part consists of 21 items querying confidence in pain management for the same vignette. Therefore, it is now possible to explore relevant factors of partial clinical competence in evidence-based decision-making for pain management, which can suggest strategies to enhance EBP competence in physical therapy. Furthermore, demographics including gender, academic degrees, and duration of clinical experience may also become relevant factors to the partial clinical competence in evidence-based decision-making for pain management considering previous studies [10,11,18]. 

The primary aim of this study was to compare the following outcomes between clinical physical therapists with and without the Cred.MDT: the partial clinical competence in evidence-based decision-making for pain management, attitudes toward biopsychosocial perspective, guideline adherence, knowledge of modern pain science, and confidence in pain management. The secondary aim was to explore relevant factors of the partial clinical competence in evidence-based decision-making for pain management.

## Materials and methods

Design

This cross-sectional study involved data analysis of (1) an anonymous paper-based survey administered to members of the Japanese Society of Allied Health and Rehabilitation as email address of each member was not known and (2) an anonymous online survey administered to members of the Japan Branch of the International McKenzie Institute, who acquired the Cred.MDT. The data collection period for each organization was two weeks. The Ethics Committee of Saitama University approved the study (No. 23100).

Participants

The inclusion criteria were (1) physical therapists and (2) individuals registered as members of the Japanese Society of Allied Health and Rehabilitation (n = 263), and members of the Japan Branch of the International McKenzie Institute with registered email addresses (n = 387) in November 2023. Exclusion criteria were individuals not currently involved in treating patients with pain, or those belonging to an education institution. Duplicates across the two cohorts were excluded.

Measures

This study included the following valid and reliable measures; (1) the PUnCQ-1 for partial clinical competence in evidence-based decision-making for pain management; (2) the part two scores of the PUnCQ for confidence in pain management; (3) the Pain Attitudes and Beliefs Scale for Physical Therapists (PABS-PT) [19] for biomedical and biopsychosocial treatment perspectives; (4) the Knowledge and Attitudes of Pain (KNAP) for knowledge of modern pain science [20]; and (5) an adherence to LBP practice guideline questionnaire [21] for an indicator of guideline adherence. Demographics included (1) gender (0 = women or 1 = men), (2) duration of clinical experience (years), (3) final academic degrees (0 = bachelor's degree or less, or 1 = post-graduate degrees in research), and (4) and acquisition of the Cred.MDT (0 = no or 1= yes). The duration of part-time clinical experience was converted to the duration of clinical experience in full-time work (40 hours/week).

The PUnCQ-1 included 12 clinical judgments for a female clinical vignette featuring whiplash-associated disorders. Each of the 12 clinical judgments includes five choices. There is one correct choice that satisfies contemporary knowledge of pain. A score of 1 is assigned for the correct response. The total score ranging from 0 to 12 was transformed into a % score. A higher % total score indicates greater clinical competence in evidence-based decision-making for pain management. Unidimensionality of the first part was confirmed in a confirmatory factor analysis with acceptable internal consistency (Cronbach’s α of 0.98) [13]. This study used the Japanese PUnCQ [22].

The PUnCQ part two has a two-factor structure in the Japanese population: (1) pain management factor (PUnCQ-2-1) with 14 items (α of 0.98) and (2) medication guidance and pain mechanism factor (PUnCQ-2-2) with 7 items (α of 0.98) [13]. The PUnCQ part two indicates confidence in pain management for the vignette. The participants rated their confidence in each management skill on an 11-point scale from 0 (not at all confident) to 10 (no problem), and the mean value was calculated for each factor. A higher mean score indicates greater confidence in each factor.

The KNAP comprises 30 items on pain physiology and attitude of management considering the biopsychosocial nature of pain. The participants rated their agreement with statements on a 6-point scale. Scores were weighted for each item [20] resulting in a total score from 0 to 150, where a higher total score indicates greater knowledge of modern pain science. Unidimensionality was confirmed with acceptable internal consistency (α of 0.8) [20]. This study used the Japanese KNAP [23].

The PABS-PT is composed of two dimensions: a biomedical treatment perspective with 10 items, and a biopsychosocial treatment perspective with nine items. The participants rated their agreement on each item with a 6-point Likert scale (1 = totally disagree, 6 = totally agree). Cronbach’s α was 0.84 for the biomedical treatment perspective domain and 0.54 for the biopsychosocial treatment perspective domain [19]. A mean value of the biopsychosocial treatment perspective domain was divided by the mean value of the biomedical treatment perspective domain (PABS-PT biopsychosocial/biomedical ratio). A higher score indicates the more biopsychosocial perspective of treatment, in which disability and pain do not have to be a consequence of tissue damage and can be influenced by psychological and social factors. This study used the Japanese PABS-PT [10].

The adherence to the LBP practice guideline questionnaire is composed of three questions for the vignette of a patient with acute nonspecific LBP without red flags [21]. The three questions concern activity, work, and bed rest. Each question has five different assertions and responses were dichotomized into guideline-inconsistent or guideline-consistent based on expert consensus [21]. The % score of guideline-consistent was calculated, where a higher % score indicates greater guideline adherence. This study used the Japanese adherence to LBP practice guideline questionnaire [10].

Analysis

The analysis was conducted using IBM SPSS Statistics for Windows, Version 28 (Released 2023; IBM Corp., Armonk, New York, United States). The level of significance was 5%. Descriptive statistics was used to understand characteristics of the participants.

First, missing values of the PUnCQ-1 and KNAP were imputed with a median value as per a previous study when the missing value was < 5% [24]. However, missing values of the PABS-PT and PUnCQ part two were not imputed because the mean value was calculated. When the missing value was > 5%, all data of the corresponding participants were excluded.

Group comparisons were conducted using the t-test for the continuous variable and the chi-square test for the binary variable. Fisher’s exact test was used when the sample was less than 10 for any category in the chi-square test. The effect size of Hedges g was computed for the continuous variable, whose interpretations were as follows: small at 0.2, moderate at 0.5, and large at 0.8. The effect size of phi was computed for the binary variable, whose interpretations were as follows: small at 0.1, moderate at 0.3, and large at 0.5. For the outcomes with a reference standard response, including the PUnCQ-1 and the adherence to the LBP practice guideline questionnaire, proportions of the reference standard response for each item were also compared between the two groups.

A multiple regression analysis was conducted using the stepwise method with the PUnCQ-1 as the dependent variable. Among the following nine independent variables, variables with a correlation coefficient of < 0.9 between the dependent variables were included in the regression analysis; (1) the PUnCQ-2-1, (2) the PUnCQ-2-2, (3) the KNAP, (4) the PABS-PT biopsychosocial/biomedical ratio, (5) the adherence to LBP practice guideline questionnaire, (6) gender, (7) duration of clinical experience, (8) final academic degrees, and (9) acquisition of the Cred.MDT. Interpretation of the R^2^ value was as follows: < 0.3, no effect or a very weak effect size; 0.3-0.5, a weak or low effect size; 0.5-0.7, a moderate effect size; and > 0.7, a strong effect size.

This study aimed to include at least 45 participants in each group, for a total of 90 participants or more. This sample size was estimated according to a previous study [10] and to satisfy the rule of > 10 participants per dependent variable for a multiple regression analysis.

## Results

Available data were collected from 122 physical therapists (59 participants from the Japanese Society of Allied Health and Rehabilitation (22.4%) and 63 participants from the Japan Branch of the International McKenzie Institute (16.3%)). Missing values consisted of one in the biopsychosocial domain of the PABS-PT and one in each of the five KNAP items, all less than 5%.

Table 1 presents the summary of the participants with the results of the group comparison. Compared to the group without the Cred.MDT, the group with the Cred.MDT had statistically greater scores with a strong effect size in the PUnCQ-2-1, KNAP, PABS-PT biopsychosocial/biomedical ratio, and adherence to the LBP practice guideline questionnaire, and with a moderate effect size in the PUnCQ-1, and PUnCQ-2-2, respectively. Furthermore, Fisher’s exact tests demonstrated a statistically significant difference in gender (p = 0.042, phi = 0.20, 17 women and 42 men among the physical therapists without the Cred.MDT, and eight women and 55 men among the physical therapists with the Cred.MDT) but no difference in the final academic degrees (p = 0.572, phi = 0.01, 53 non-post-graduate and six post-graduate degrees among the physical therapists without the Cred.MDT, and 57 non-post-graduate and six post-graduate degrees among the physical therapists with the Cred.MDT) between the two groups. Table 2 shows comparisons of correct response percentages between the two groups on each item of the PUnCQ-1. Although effect sizes of all items with a statistically significant difference were small, physical therapists with the Cred.MDT had higher correct responses on Item 3, ‘making an objective measure of her (the vignette’s) pain by seeing how much damage there is on magnetic resonance image (MRI)’, Item 5, ‘checking for yellow flags’, Item 9, ‘helping her understand her pain’, Item 11, ‘doing further investigations’, and Item 12, ‘supporting her to use self-management techniques to manage her pain’. Table 3 shows comparisons of guideline-consistent response percentages between the two groups on each item of the adherence to LBP practice guideline questionnaire. In each item, greater guideline-consistent % was detected in the physical therapists with the Cred.MDT than those without it.

**Table 1:** Summary of the participants and differences between the two groups. Values are expressed with mean (standard deviations). An absolute value was presented in the effect size. PUnCQ, the Pain Understanding and Confidence Questionnaire.

Variable	Japanese Society of Allied Health and Rehabilitation group (n = 59)	Japan Branch of the International McKenzie Institute group (n = 63)	p-value	Hedges g (95% confidence intervals)
PUnCQ part one (%)	57.6 (12.1)	64.9 (13.1)	0.002	0.58 (0.22 to 0.94)
PUnCQ part two pain management factor (0−10)	4.8 (2.0)	6.3 (1.9)	<0.001	0.80 (0.44 to 1.17)
PUnCQ part two medication guidance and pain mechanism factor (0−10)	4.0 (2.1)	5.1 (2.1)	0.003	0.55 (0.19 to 0.91)
Knowledge and Attitudes of Pain (0−150)	78.2 (4.8)	84.3 (6.1)	<0.001	1.10 (0.72 to 1.48)
Pain Attitudes and Beliefs Scale for Physical Therapists biomedical treatment perspective (1−6)	4.2 (0.5)	3.5 (0.7)	<0.001	1.15 (0.76 to 1.52)
Pain Attitudes and Beliefs Scale for Physical Therapists biopsychosocial treatment perspective (1−6)	3.7 (0.5)	4.3 (0.6)	<0.001	1.05 (0.67 to 1.42)
Pain Attitudes and Beliefs Scale for Physical Therapists biopsychosocial/biomedical ratio	0.9 (0.2)	1.3 (0.4)	<0.001	1.39 (0.99 to 1.78]
Adherence to low back pain practice guideline (%)	46.3 (29.7)	79.9 (25.8)	<0.001	1.20 (0.82 to 1.58)
Clinical experience (year)	10.2 (7.9)	13.3 (6.3)	0.014	0.45 (0.09 to 0.80)

**Table 2:** Comparisons of correct response percentages between the two groups on each item of the Pain Understanding and Confidence Questionnaire part one (PUnCQ-1). *Fisher’s exact test

Items	Japanese Society of Allied Health and Rehabilitation group (n = 59)	Japan Branch of the International McKenzie Institute group (n = 63)	p-value	Phi
Item 1: Changing her painkillers	69.5	54.0	0.078	0.16
Item 2: Starting medication for neuropathic pain	57.6	54.0	0.684	0.04
Item 3: Making an objective measure of her pain by seeing how much damage there is on Magnetic Resonance Image	8.5	30.2	0.002*	0.27
Item 4: Encouraging her to do more exercise	16.9	22.2	0.464	0.07
Item 5: Checking for yellow flags	74.6	93.7	0.004*	0.26
Item 6: Checking for red flags	61.0	55.6	0.541	0.06
Item 7: Asking the patient to rate the severity of their pain	69.5	82.5	0.091	0.15
Item 8: Starting an anti-depressant	59.3	63.5	0.636	0.04
Item 9: Helping her understand her pain	86.4	100.0	0.002*	0.27
Item 10: Referring to a psychologist	83.1	81.0	0.763	0.03
Item 11: Doing further investigations	25.4	46.0	0.018	0.21
Item 12: Supporting her to use self-management techniques to manage her pain.	79.7	96.8	0.003*	0.27

**Table 3:** Comparisons of guideline-consistent response percentages between the two groups on each item of the adherence to low back pain practice guideline questionnaire. *Fisher’s exact test

Items	Japanese Society of Allied Health and Rehabilitation group (n = 59)	Japan Branch of the International McKenzie Institute group (n = 63)	p-value	Phi
Item 1: Activity	76.3	93.7	0.010*	0.25
Item 2: Work	39.0	71.4	<0.001	0.33
Item 3: Bed rest	23.7	74.6	<0.001	0.51

In relation to the multiple regression analysis, there was no variable whose correlation coefficients were > 0.9 (Table 4). Notably, a statistically significant positive correlation was detected between the PABS-PT biopsychosocial/biomedical ratio and male gender. Consequently, the PUnCQ-2-1 and acquisition of the Cred.MDT statistically significantly contributed to the PUnCQ-1 (Table 5). The R^2^ values of 0.12 indicated no effect or a very weak effect size. The Durbin-Watson statistic was 1.86. There was no outlier for which the predicted value of the measured value was above 3 SD.

**Table 4:** Pearson’s correlation coefficients between variables. PUnCQ, Pain Attitudes and Beliefs Scale for Physical Therapists; PABS-PT, Pain Attitudes and Beliefs Scale for Physical Therapists; Cred. MDT, credential license in the McKenzie method of Mechanical Diagnosis and Therapy. Gender: 0: women; 1: men Final academic degrees: 0: bachelor's degree or less; 1: post-graduate degrees in research Acquisition of the Cred.MDT: 0: without the Cred.MDT; 1: with the Cred.MDT *p<0.05; **p≤0.01

Variables	1	2	3	4	5	6	7	8	9	10
PUnCQ part one	1.00	0.30^**^	0.30^**^	0.24^**^	0.17	0.23^**^	-0.06	0.21^*^	0.06	0.28^**^
PUnCQ part two pain management factor		1.00	0.84^**^	0.41^**^	0.36^**^	0.32^**^	0.23^**^	0.36^**^	0.05	0.38^**^
PUnCQ part two medication guidance and pain mechanism factor			1.00	0.30^**^	0.18	0.27^**^	0.15	0.34^**^	0.09	0.27^**^
Knowledge and Attitudes of Pain				1.00	0.63^**^	0.44^**^	0.28^**^	0.29^**^	0.07	0.49^**^
PABS-PT biopsychosocial/biomedical ratio					1.00	0.40^**^	0.26^**^	0.16	-0.02	0.58^**^
Adherence to low back pain practice guideline						1.00	0.18^*^	0.33^**^	<0.01	0.52^**^
Gender							1.00	0.08	0.17	0.20^*^
Duration of clinical experience								1.00	0.10	0.22^*^
Final academic degrees									1.00	0.01
Acquisition of the Cred.MDT										1.00

**Table 5:** Results of a multiple regression model for the Pain Understanding and Confidence Questionnaire (PUnCQ) part one scores.

Model	Unstandardized coefficients (B)	95% confidence intervals	Standardized coefficients (β)	p-value (95% confidence intervals)
(Constant)	50.90	44.60–57.19		<0.001
PUnCQ part two pain management factor	1.41	0.27–2.56	0.23	0.016
Acquisition of the credential license in the McKenzie method of Mechanical Diagnosis and Therapy (0 = without, 1 = with)	5.08	0.29–9.87	0.19	0.038

## Discussion

This study examined whether clinical physical therapists with and without the Cred.MDT differed in terms of the partial clinical competence in evidence-based decision-making for pain management, attitudes toward biopsychosocial perspective, guideline adherence, knowledge of modern pain science, and confidence in pain management. Consequently, in all of these, clinical physical therapists with the Cred.MDT were shown to be better with an effect size of moderate or higher than those without it. Some results coincide with the results of a previous study [10], and this is not surprising given that the educational curriculum of MDT has been constantly updated with the latest evidence. In particular, a detailed analysis of the PUnCQ-1 found that clinical physical therapists with the Cred.MDT had higher correct responses on five items than those without it; ‘making an objective measure of her pain by seeing how much damage there is on MRI’, ‘checking for yellow flags’, ‘doing further investigations’, ‘helping her understand her pain’, and ‘supporting her to use self-management techniques to manage her pain’. The first three items were related to biopsychosocial perspectives, consistent with the group differences found in the PABS-PT biopsychosocial/biomedical ratio. With regard to the last two items, these may reflect that MDT places an emphasis on self-management. These five items in which group differences were observed are consistent with the 11 concepts of optimal musculoskeletal management [25]. Therefore, obtaining the Cred.MDT may be a beneficial process for physical therapists to increase their management skills for dealing with musculoskeletal problems. However, at this time, it is unclear whether MDT is the best system for enhancing pain management skills, and future comparisons with training in other effective biopsychosocial approaches including the cognitive functional therapy are needed [26]. Understanding the applicability and limitations of each system will contribute to better life-long education for clinical physical therapists, which will ultimately lead to better medical care for patients.

Interestingly, correct responses to item 3 and item 4 were low in both groups. Since the PUnCQ has not yet been compared internationally, we do not know whether this suggests a characteristic of Japanese physical therapists or whether the content requires further education internationally. Especially for imaging findings such as MRI, some countries are relatively easy to obtain due to differences in social security systems, while other countries are not so easy. Therefore, an international comparison of the PUnCQ may reveal such differences. Item 4 concerns exercise therapy for chronic pain. This research area is evolving daily, and item 4 may also change as the EBP develops as a framework for clinical decision-making has been recently proposed [27]. In any case, the data from this study indicate a need for further education in this area.

Regarding the guideline adherence, greater guideline-consistent % were detected in the physical therapists with the Cred.MDT than those without it. Interestingly, the guideline-consistent % appears to have not changed since 2014 among physical therapists with the Cred.MDT (guideline-consistent in activity = 95%, guideline-consistent in work = 77%, and guideline-consistent in bed rest = 70% in 2014) [10]. On the other hand, the guideline-consistent % appears to have not increased since 2014 among physical therapists without the Cred.MDT (guideline-consistent in activity = 74%, guideline-consistent in work = 57%, and guideline-consistent in bed rest = 20% in 2014), rather the guideline-consistent % decreased [10]. The fact that the guideline-consistent % has not changed over the last decade is consistent with the findings of a previous study that physical therapists' attitudes toward EBP have not changed over the last five years in Japan [3]. Furthermore, the decrease in the guideline-consistent % for work may reflect the fact that the percentage of physical therapists who are treating patients as recommended by the guidelines has declined from 50% to 35% over the past decade [2]. These results indicate a need for advancing specific strategies to promote EBP that are supported by clinical physical therapists regardless of whether they have the Cred.MDT or not [28].

Evidence-based clinical decision-making should take into account the patient's overall biopsychosocial status, medical history, and thinking. The fact that the PUnCQ-1 had positive but weak correlation coefficients with the KNAP and PABS-PT biopsychosocial/biomedical ratio in the cross-correlation analysis indicates that clinical competence in evidence-based clinical decision-making for pain management is not measured by assessing respondent’s knowledge or attitudes. Thus, the PUnCQ-1, which selects the correct response by considering these of the vignettes, is a unique measure, and this study explored its relevant factors using the multiple regression analysis. Consequently, the PUnCQ-2-1 and acquisition of the Cred.MDT were statistically significant contributing factors. The relevant factor of the acquisition of the Cred.MDT makes sense from the results of the group comparisons above. Interestingly, the therapists' subjective confidence in pain management shown with the PUnCQ-2-1 associated with the partial clinical competence in evidence-based decision-making for pain management shown with the PUnCQ-1. Such a clinical competence is not easily measured and cannot be constantly monitored to visualize one's progress. On the other hand, subjective confidence can be easily monitored over time. Therefore, during daily patient care, checking the PUnCQ-2-2 items may provide some indication of progress in evidence-based clinical decision-making competence.

In relation to gender, the male factor was positively associated with attitude toward biopsychosocial approach. According to a previous review on physicians [18], women do more psychosocial counseling than men, and therefore, we expected female physical therapists to have a higher attitude toward biopsychosocial approach than male physical therapists, but the opposite finding was revealed in the present study. Simply, a reason for the difference may be due to the difference between the physicians and physical therapists. However, to the best of the authors' knowledge, studies examining the influence of sex/gender on attitude toward biopsychosocial approach among physical therapists are limited, and the results of this study provide a preliminary finding for further research in this area. More importantly, although demographically speaking, gender differences among physical therapists in Japan are not large, the research participants of women was smaller than those of men, which is consistent with the results of previous studies with different populations [10,11]. This may be a unique trend in Japan. There were also differences in gender proportions between the two groups in this study. These findings may be associated with the fact that attitudes toward research participation correlate with the ability to have an attitude toward EBP [3]. According to a 2010 national survey of Japanese female physical therapists, 44.5% of the respondents indicated that they had experienced discrimination with regard to their appraisal or promotion, and 34% of them preferred to work shorter hours when returning to work after leaving their jobs [29]. Improving the system so that women can receive more recurrent education may be required in Japan.

Limitations

There are some limitations to this study. The first limitation is the lack of post-graduate educational factors. In Japan, there is no system of clinical master's degrees, and therefore, the academic degrees in this study do not include clinical master's degrees. Furthermore, the orthopedic manual physical therapy (OMPT) is considered a clinical standard for musculoskeletal physical therapy in the world, but this study did not examine the effect of having or not having the OMPT. Therefore, the impact of clinical master's degrees and the acquisition of the OMPT on the clinical decision-making competence should be examined in cultures where there are a large number of those holders. Second, because the PUnCQ uses vignettes featuring whiplash-related impairments, and the PABS-PT and the adherence to LBP practice guideline questionnaire use situations related to LBP, it is not certain if these measures reflect their corresponding abilities in all musculoskeletal disorders. Third, the regression model for the PUnCQ-1 showed that two factors were statistically significant contributors, but this model had just no effect or a very weak effect size. Therefore, there may be major contributing factors other than the outcomes used in this study. For example, attitudes toward EBP may be relevant [30]. It would be important to analyze with a broader range of relevant factors, especially with modifiable factors, in order to develop strategies to promote EBP in the future. Finally, data collection methods were different between the two group, which might affect the results. However, the response rates are not substantially different (22.4% in the Japanese Society of Allied Health and Rehabilitation and 16.3% in the Japan Branch of the International McKenzie Institute), and we believe that differences in data collection methods have had negligible impact on the results of this study.

## Conclusions

This study found that clinical physical therapists with the Cred.MDT have greater clinical competence in evidence-based decision-making for pain management, more attitudes toward the biopsychosocial perspective, greater guideline adherence, greater knowledge of modern pain science, and greater confidence in pain management than those without the Cred.MDT. Confidence in pain management and acquisition of the Cred.MDT contributed to the clinical competence in evidence-based decision-making for pain management. This study provides evidence of MDT as an effective post-graduate educational program to promote EBP in physical therapy. Further research is needed to develop other strategies for training quality physical therapists.
